# Good reintegration of unstable osteochondritis dissecans at the femoral condyle following screw fixation: A 3‐year magnetic resonance imaging and clinical follow‐up study

**DOI:** 10.1002/jeo2.70331

**Published:** 2025-07-13

**Authors:** Franziska L. Breulmann, Patrice Dominitz, Alexander Otto, Sebastian Siebenlist, Julian Mehl

**Affiliations:** ^1^ Department of Sports Orthopaedics, Klinikum Rechts der Isar Technical University of Munich Munich Germany

**Keywords:** cartilage regeneration, knee, osteochondral defects, osteochondritis dissecans

## Abstract

**Purpose:**

Osteochondritis dissecans (OD) is characterized by detachment of osteochondral fragment. Surgical refixation is recommended in unstable lesions. The aim of present study was to analyse the reintegration of the cartilage after screw fixation of unstable OD in the knee using magnetic resonance imaging (MRI).

**Methods:**

A total of 16 cases were retrospectively included in the present study. At a minimum follow‐up of 1 year, MRI was performed, and the cartilage quality was quantified using the Morphological Cartilage Assessment and Reporting Tool (MOCART). Clinical outcomes were evaluated at the same time point as the MRI assessment using the visual analog scale (VAS), the subjective knee evaluation form of the International Knee Documentation Committee (IKDC), the Knee Osteoarthritis Outcome Score (KOOS) and the return to sports (RTS) rate.

**Results:**

The average age was 18.6 ± 7.4 years. Thirteen lesions (81.2%) were located on the medial femoral condyle, while three lesions (18.7%) were found on the lateral femoral condyle. At a mean follow‐up of 47.5 ± 24.6 months, evaluation of the MRIs showed an average MOCART score of 64.4 ± 17.8 indicating good cartilage condition. The mean IKDC score was 80.1 ± 14.9, the median VAS for pain was 0 (interquartile range 0–3), and the mean results of the KOOS were as follows: KOOS symptoms 84.6 ± 12.7, KOOS pain 87.7 ± 10.7, KOOS activities of daily living 95.6 (91.8–98.9), KOOS sport and recreation function 85.0 (60.0–90.0) and KOOS knee‐related quality of life 75.0 (60.9–81.3). All the patients were able to RTS activities postoperation.

**Conclusion:**

Surgical screw refixation of unstable OD at the femoral condyles yielded good cartilage integration in MRI follow‐up and excellent clinical outcomes as well as high RTS rates at a mid‐term follow‐up.

**Level of Evidence:**

Level II.

**Abbreviations**
ACI: autologous chondrocyte implantation
ICRS: International Cartilage Repair Society
IKDC: International Knee Documentation Committee
IQR: interquartile range
KOOS: knee osteoarthritis outcome score
MACT: matrix‐associated autologous chondrocyte transplantation
MOCART: Morphological Cartilage Assessment and Reporting Tool
MRI: magnetic resonance imaging
OD: osteochondrosis dissecans
PROM: patient‐reported outcome measures
ROM: range of motion
RTS: return to sports rate
SD: standard deviation
TAS: Tegner activity scale
VAS: visual analog scale

## INTRODUCTION

Osteochondritis dissecans (OD) is a condition involving subchondral bone and cartilage changes, potentially leading to the detachment of osteochondral fragments [16]. With an incidence of 15.4 per 100,000 in males and 3.3 per 100,000 in females between the age of 6 and 19 years, OD is a relatively rare disease [9]. However, OD can lead to a severely limited joint function and increased risk for early osteoarthritis [4, 12, 17]. The knee joint is the most commonly affected location in OD lesions [11, 12, 30], with the medial femoral condyle being the most frequently observed site (66.2%), followed by the lateral femoral condyle (18.1%) [22].

The exact aetiology of OD remains unclear, but discussions often highlight the potential role of recurrent microtrauma, vascular, hormonal, dysplastic and genetic factors [30, 34]. Additionally, increased stress in the affected joint, such as from malalignment or high‐impact sports, may contribute to OD development at the knee [6, 30].

From a pathophysiological perspective, OD involves aseptic necrosis progressing through several stages. The common International Cartilage Repair Society (ICRS) classification defines four grades of OD in the knee joint [3]. In the early stages, the subchondral bone becomes necrotic but remains stable, while later stages see the formation and gradual detachment of an osteochondral fragment until it becomes a free joint body [9, 14].

Treatment options are mostly dependent on the stability of the OD fragment. For stable lesions, conservative treatment is initially attempted [13], which includes weight‐bearing restriction of the affected joint and avoiding triggering stimuli like high‐impact sports [1, 5]. In case of persistent symptoms, stable lesions can also be treated by subchondral drilling [7]. In the case of symptomatic unstable lesions, surgery is commonly recommended [7]. In the event of an unstable and unsalvageable lesion or unsuccessful refixation, the free joint body should be removed, followed by reconstruction of the defect area using cartilage regenerative techniques [7]. For unstable lesions with intact cartilage surface, a refixation of the osteochondral fragment may be considered. Thus, the original hyaline cartilage can be preserved. However, there are concerns regarding the healing potential as OD is characterized by limited blood supply and subsequent necrosis. Even though previous studies showed good clinical outcomes after OD refixation [15, 35], there are no studies presenting the MR morphological results. The primary advantage of refixation is the anatomical reconstruction of the joint surface with the patient's own cartilage. This method ensures the preservation and reintegration of the original cartilage into the joint, thereby maintaining its natural structure and function to delay or prevent the onset of premature osteoarthrosis [31].

The aim of present study was to investigate the outcomes of OD refixation at the femoral condyle and to analyse the reintegration of the fragment by means of magnetic resonance imaging (MRI). It was hypothesized that surgical OD refixation using metal screws leads to good MR morphologic as well as to good clinical results and high return to sport (RTS) rates at a mid‐term follow‐up. Our study is about to fill this gap by evaluating the short‐ to mid‐term outcomes of this surgical approach and providing insights into the efficacy of screw fixation for restoring cartilage and joint function in these cases.

## METHODS

This monocentric retrospective study was approved by the Ethical Committee of the Technical University of Munich (2022‐678‐S‐SR). A search of the institutional database was conducted to identify patients with unstable (ICRS stage III or IV) OD who underwent surgical therapy with screw fixation of the fragment. The patients underwent surgery at an age range of 12–55 years, with operations taking place between January 2010 and August 2020.

Patients with a follow‐up of less than 1 year were excluded. Further exclusion criteria were concomitant rheumatic diseases, as well as systemic or dystrophic soft tissue diseases, diabetes mellitus, systemic neurological diseases and psychiatric disorders. Patients who had undergone previous surgical procedures on the menisci, cruciate ligaments, collateral ligaments, or tibio‐femoral cartilage were also excluded. Furthermore, patients with previous fractures or osteotomies of the lower extremities were excluded from the study.

Informed consent was obtained from each patient and the patients included were contacted exclusively for the purpose of this study, part of the subject population participated in previous investigations at this institution.

### Indications

Patients were selected for OD refixation if they exhibited symptomatic OD in an unstable stage III or IV according to ICRS, and if the fragment showed vital cartilage during arthroscopy examination. In addition to previously mentioned exclusion criteria, refixation was contraindicated if the fragment itself was broken or the cartilage of the fragment showed relevant damage (>ICRS grade 2).

### Surgical technique

**Figure 1:**
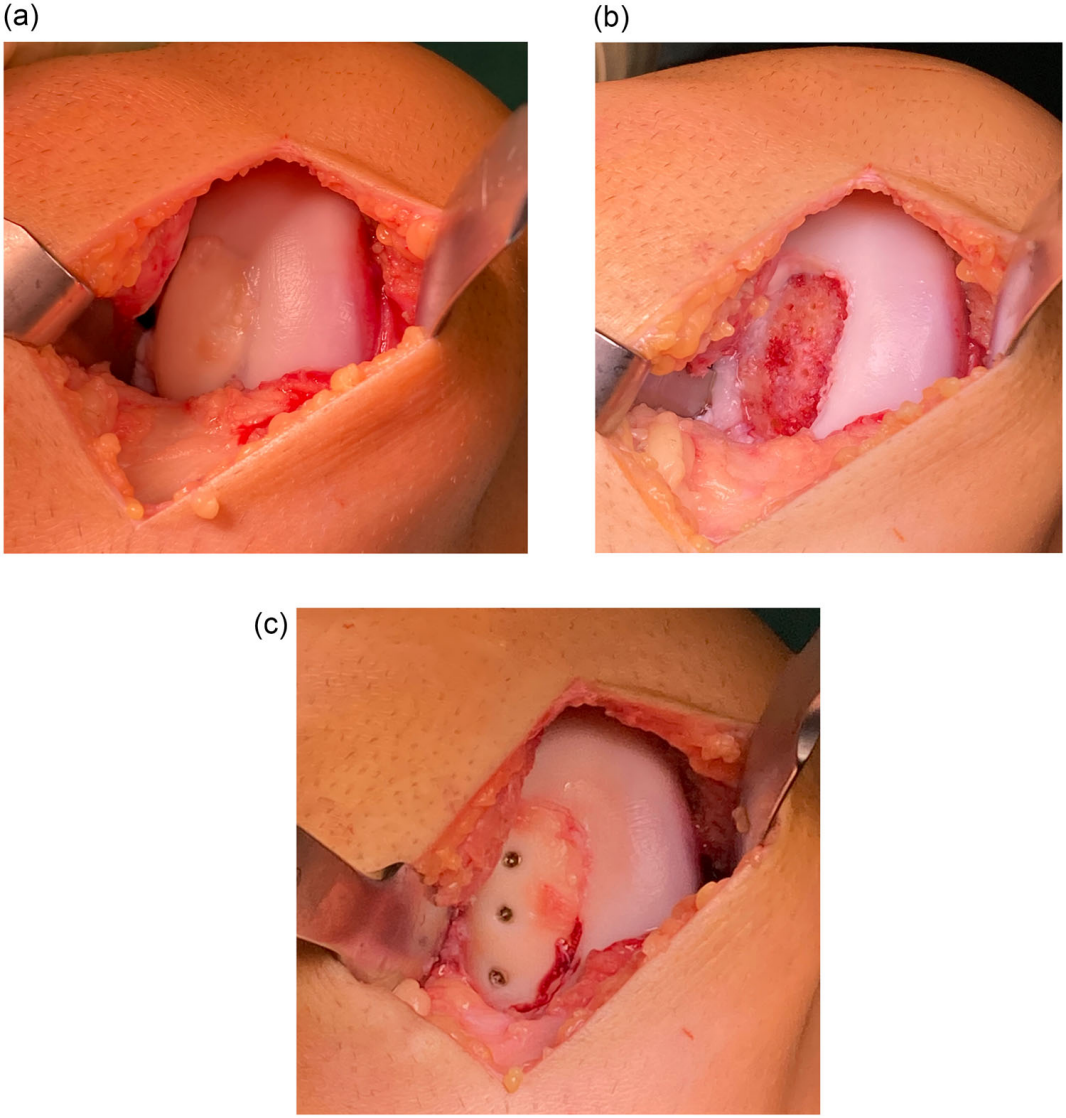
(a) Unstable osteochondrosis dissecans (OD) fragment in situ (medial femoral condyle of the right knee). (b) Defect bed after debridement and drilling of the sclerotic bone. (c) OD fragment after fixation using three 2 mm mini‐AO screws.

The OD fragment was arthroscopically assessed for integrity, as well as vitality and size. The refixation of the OD fragment was performed with a mini‐open arthrotomy (Figure 1).

In the event that the fragment was still attached to the surrounding tissue, it was secured in situ. Conversely, in instances where the fragment has already been detached, the preparation of the bone bed was undertaken. In four cases, an additional bone grafting with autologous cancellous bone was performed due to subchondral bone sclerosis or insufficient defect coverage of the bony fragment bed. After debridement of the fragment bed, the fragment was replaced in position and refixed with small fragment screws. The number of screws was dependent on the size and shape of the fragment. Mini‐AO‐Screws with a diameter of 1.5–2.0 mm were utilized also depending on the fragment size. The length of the screws was tailored to the specific dimensions and bone characteristics of the patient.

### Postoperative rehabilitation

Postoperatively, the patients were not allowed to put any weight on the affected lower limb for 6 weeks. After 6 weeks, the screws were arthroscopically removed in a second procedure, in four cases a mini‐open approach was necessary. Afterward, weight bearing was gradually increased after radiographic control until the patients return to full weight bearing after around 2 weeks after the second surgery. Range of motion (ROM) was unlimited directly after the primary surgery. At 3 months, RTS and work was recommended for low‐impact activities, and at 9 months for high‐impact activities. Postoperative rehabilitation was adapted if concomitant procedures were performed at index surgery.

### MRI assessment

All patients underwent MRI of the affected knee on average 47.5 ± 24.6 months after the operation. Two independent experts conducted a qualitative assessment of the MRI images using the Morphological Cartilage Assessment and Reporting Tool (MOCART) scoring system to evaluate the reintegration of the osteochondral fragment. The MOCART 2.0 knee score comprises a range of evaluation criteria for assessing the cartilage defect, as well as the subchondral bone. The integration was evaluated, as well as the surface for integrity and any irregularities. Bone alterations may manifest as defects or overgrowth and the condition of the subchondral lamina, as well as the presence of oedema, cysts, or osteonecrosis‐like signals were described [28]. MRI evaluations were independently performed by an assistant physician and a doctoral student to assess interobserver reliability.

MRI scans were performed using a 3T Siemens Magnetom Verio MRI machine. The imaging protocol included several key sequences to provide a comprehensive assessment of the osteochondral defects. These sequences included sagittal T1‐and T2‐weighted turbo spin echo (TSE), sagittal proton density (PD) with fat suppression (FS), coronal PD with FS, and transverse PD with FS. There were no significant differences in the imaging protocol across different scanning sessions.

### Clinical outcome parameters

Patient‐reported outcome measures (PROMs) were collected as well at an average follow‐up of 47.5 ± 24.6 months postoperatively. Patients completed questionnaires that captured postoperative and current symptoms, as well as the assessment of current pain using the visual analog scale (VAS). The evaluation of knee function was carried out using the subjective knee evaluation form of the International Knee Documentation Committee (IKDC) and the Knee Osteoarthritis Outcome Score (KOOS). The Tegner Activity Score was employed to assess the activity levels of the patients [10]. Additionally, the patients were asked for any postoperative complications (infection, problems of wound healing, stiffness) or revision procedures beyond the removal of the screws.

### RTS

To evaluate RTS, the patients reported their specific preoperative (1 year prior to surgery) and postoperative (final follow‐up) participation in 34 different sporting activities. Parameters included the level of sport, frequency of participation and duration of each session. The types of sports were categorized by low, intermediate, high impact and timing of RTS. Additionally, timing of return to the current level of sports at final follow‐up and qualitative change of sporting ability were evaluated. The patients were also asked to rate their current function of the leg (excellent, good, satisfactory, and bad).

### Clinical evaluation

A thorough clinical examination was conducted on the affected joint by a single examiner. The assessment focused on various clinical aspects, including signs of inflammation such as swelling, joint effusion and tenderness. Ligamentous stability of the knee was evaluated through standardized manual stability tests, including the Lachman test, anterior and posterior drawer tests and varus/valgus stress tests. Additionally, the ROM was carefully assessed using a goniometer to ensure precise measurement of knee flexion and extension.

### Statistical analysis

Descriptive statistics were presented as counts and percentages for categorical variables. Continuous variables were assessed for normality using the Shapiro–Wilk test and reported as mean ± standard deviation (SD) or median and interquartile range (IQR), depending on the distribution. Descriptive statistical analyses, including calculations of means, SDs and normality testing, were performed using Microsoft® Excel for Mac [19]. Interobserver reliability was assessed using the intraclass correlation coefficient (ICC, model 2,1), computed via a mixed‐effects model, treating cases as random effects. Additionally, Pearson correlation was calculated to evaluate the linear relationship between raters, and root mean squared error (RMSE) was used to quantify the mean deviation between scores. These statistical analyses were conducted using Python 3.9 [25], utilizing the statsmodels [29], SciPy [32] and scikit‐learn [23] libraries.

## RESULTS

In total, 15 patients (seven females and eight males) were included. One male patient underwent surgery on both knees. For convenience, both knees were considered as separate cases below. In our study, 13 lesions (81.2%) were located on the medial femoral condyle, while three lesions (18.7%) were found on the lateral femoral condyle. At the time of surgery, six patients still had open growth plates. Further characteristics of the study cohort are presented in Table 1.

**Table 1:** Patients' demographics.

Number of cases (*N*)	16
Sex (male/female)	9/7 (56.2% male)
Body mass index (kg/m^2^)	23.4 ± 5.0
Age at time of surgery (years)	18.6 ± 7.4
Follow‐up (months)	47.5 ± 24.6
Defect size	3.95 ± 2.41 cm²

*Note*: Normally distributed continuous variables are shown as means ± standard deviation. Categorical variables are shown as percentages.

### MRI assessment

**Figure 2:**
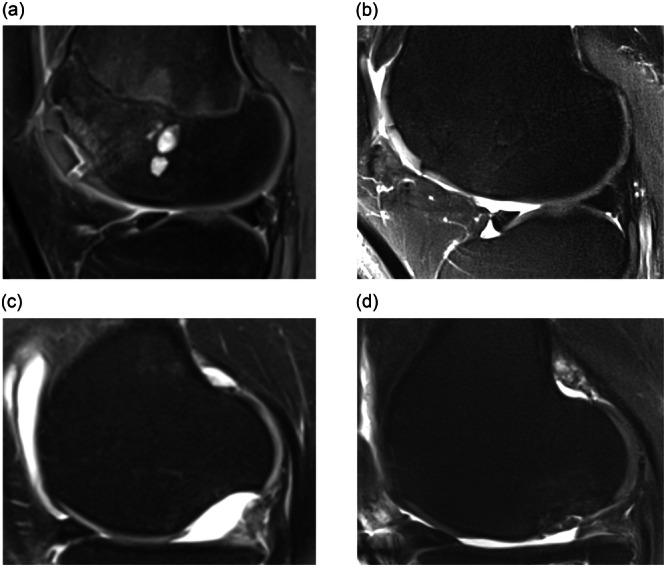
Representative magnetic resonance imaging images illustrating preoperative (a) and postoperative (b) findings for Patient 1, showing complete integration of the lesion on the lateral femoral condyle at 75 months postsurgery. Preoperative (c) and postoperative (d) images for Patient 2 demonstrate incomplete integration of the lesion on the medial femoral condyle at 44 months postsurgery.

The evaluation of cartilage repair using the MOCART scoring system yielded an overall average Score of 64.4 ± 17.8 out of a possible 100 points (Figure 2). The results of the individual subcategories can be found in Table 2.

**Table 2:** Results of the Morphological Cartilage Assessment and Reporting Tool 2.0 score.

Subcategory	Mean ± SD	Maximum achievable points
Volume fill of cartilage defect	14.8 ± 5.2	20
Integration into adjacent cartilage	11.9 ± 4.2	15
Surface of the repair tissue	5.0 ± 2.9	10
Structure of the repair tissue	4.4 ± 4.7	10
Signal intensity of the repair tissue	12.0 ± 2.5	15
Bony defect or bony overgrowth	4.7 ± 2.9	10
Subchondral changes	11.6 ± 7.8	20
Total	64.4 ± 17.8	100

### Clinical outcome

The results of the PROMs demonstrated favourable outcomes, with most results falling within the ‘good’ to ‘very good’ range (Table 3).

**Table 3:** Patient‐reported outcome measures.

VAS for pain	0.0 (0.0–3.0)
TAS	5.0 ± 2.3
IKDC	80.1 ± 14.9
*KOOS* symptoms	84.6 ± 12.7
*KOOS* pain	87.7 ± 10.7
*KOOS* activities of daily living	95.6 (91.8–98.9)
*KOOS* sport and recreation function	85.0 (60.0–90.0)
*KOOS* knee‐related quality of life	75.0 (60.9–81.3)

*Note*: Normally distributed continuous variables are shown as means ± standard deviation. Nonnormally distributed continuous variables are shown as median (25–75%) interquartile range.

Abbreviations: IKDC, International Knee Documentation Committee subjective knee form; KOOS, knee injury and osteoarthritis outcome score; TAS, Tegner activity scale; VAS, visual analog scale.

### RTS

The mean time to RTS was 7.0 ± 3.7 months. It is noteworthy that all individuals who were engaged in sports prior to surgery were able to resume their athletic activities postoperatively. Upon closer examination of the specific sports and their respective intensities, some patients elected to modify the type of sport they engaged in, while others reduced the number of weekly hours dedicated to their previously pursued sports activities.

### Clinical evaluation

The median ROM (flexion/extension) among the subjects was 147.5‐0‐0 degrees. The examination revealed no evidence of inflammatory changes. The stability of the medial and lateral collateral ligaments was examined, and no evidence of instability or pathologic motion was noted. The examination of the cruciate ligaments also showed unremarkable results. The presence of pain or trigger points was examined. Two patients reported mild pain with maximum flexion and two others reported pain with pressure on the joint space.

### Complications

One patient underwent a second surgical procedure following the twisting of their knee during the healing phase, which resulted in the loosening of the fragment. In another patient, the procedure did not lead to a satisfactory result. The image morphological findings indicated insufficient healing of the fragment. In the presence of a leg axis malalignment, the patient was advised to undergo a realignment osteotomy.

### Interobserver reliability

The interobserver reliability analysis for the MOCART score showed an ICC of 0.85, indicating good agreement between the raters. The Pearson correlation was 0.88, demonstrating a strong linear relationship, while the RMSE was 10.0 MOCART points, suggesting moderate observer variability. For cases with strongly divergent ratings, a consensus assessment was performed.

## DISCUSSION

The results of present study illustrate that refixation of unstable OD using metal screws leads to good reintegration of the fragment with an average MOCART score of 64.4 ± 17.8. Additionally, favourable clinical results were achieved, with an IKDC score of 80.1 ± 14.9 and a Tegner activity scale (TAS) score of 5.0 ± 2.3. Furthermore, high return‐to‐sport rates (RTS rate: 100%) and low complication and reoperation rates were observed.

The inclusion of adolescent patients (37.5% of all patients) in this study reflects the fact that OD predominantly affects younger individuals. Given that the mean patient age in this cohort was 18 years, most cases were still within the adolescent or young adult range. The biological healing potential in adolescents is known to be higher compared to adults, as the cartilage and subchondral bone are still in a phase of active remodelling. This may influence the reintegration of refixed OD fragments and lead to better outcomes in younger patients. It is important to acknowledge that OD in adolescents differs from degenerative OD in older adults, which is often associated with pre‐existing cartilage damage and subchondral changes. While this study did not specifically differentiate outcomes between adolescents and older patients, the overall positive results suggest that screw fixation can be effective across different age groups. Future studies with larger cohorts should investigate whether age‐related biological factors influence long‐term cartilage integration and clinical outcomes.

In a systematic review conducted by Matthews et al. [18], the outcomes of 24 studies on various treatment options for unstable OD were examined. The follow‐up period for these studies ranged from 2 to 17 years. They reported an IKDC range of 75–85 and a Tegner Score of 4 to 5. The results of the present study can be considered favourable in comparison. Since we included an age range up to 55 years old, with a mean age of 18 years, some patients might report a lower Tegner score or show a less good integration of the fragment due to the age and possible degenerative changes [21]. Rüther et al. [26] examined refixation using bioresorbable polylactide implants and reported a MOCART score of 55 after an average follow‐up of 13.9 years. In comparison, our study achieved higher values (64.4), supporting the efficacy of metal screws.

In a study of matrix‐associated autologous chondrocyte transplantation (MACT) for osteochondral defects of the knee, Andriolo et al. [2] reported favourable clinical and radiological results, with an average IKDC score of 85 points and a MOCART score of 72.9 points. While these scores are slightly higher than the average MOCART and IKDC score observed in present study, the outcomes of refixation remain competitive, particularly given the advantage of using the patient's own hyaline cartilage.

Sacolick et al. [27] assessed the efficacy of autologous chondrocyte implantation (ACI) for the treatment of osteochondral lesions in adult patients. The findings indicated a notable improvement in symptoms, with an average IKDC score of 82.7. The study reported a relevant complication rate of 15.6% and a failure rate of 8.2%. In comparison, the present study observed a low rate of complications and treatment failure, suggesting that refixation of unstable OD fragments may offer a more straightforward and lower‐risk alternative to cell‐based cartilage repair techniques. While both approaches lead to satisfactory clinical outcomes, the advantage of maintaining the patient's original hyaline cartilage in refixation may contribute to a more predictable healing process with fewer postoperative complications.

A systematic review was conducted by Vivekanantha et al. [33], in which the results of studies examining various treatment options for both stable and unstable OD were analysed. An RTS rate of 93.3% was reported. Therefore, the RTS rate of 100% observed in the present study can be considered favourable.

Nammour et al. emphasize the significance of fragment stability in the efficacy of OD treatments. Metallic screws, which are widely recognized for their mechanical robustness, ensure firm fixation, thereby minimizing the risk of fragment displacement. The authors emphasize that these screws provide reliable compression. In comparison, bioresorbable implants, while avoiding a second procedure for hardware removal, present challenges such as implant degradation, breakage and inflammatory responses, which could compromise long‐term outcomes. These findings support the efficacy of metallic screws for achieving favourable reintegration and clinical results [20].

The findings of Perelli et al. [24] indicate that refixation of OD lesions in skeletally mature patients yields consistently favourable long‐term outcomes. No significant differences were observed in functional scores (mean IKDC: 79, mean Lysholm: 83) or radiographic healing rates (74%) when comparing different fixation devices, including metallic screws, Herbert screws and bioabsorbable nails. It is noteworthy that complete stability of the osteochondral fragments was confirmed in all cases during second‐look arthroscopies, even when radiographic consolidation was incomplete. Nevertheless, superior functional outcomes were associated with radiographic union, highlighting the critical role of achieving radiographic consolidation in ensuring the success of the treatment and the long‐term stability of the osteochondral fragment. This emphasizes that radiographic healing is an important indicator of treatment efficacy, as it correlates with better functional recovery and a lower risk of subsequent complications.

In the event that refixation is not feasible or unsuccessful, alternative reconstructive procedures can be undertaken at a subsequent point in time as a secondary option. Therefore, refixation can be regarded as the initial therapeutic strategy for unstable OD, while leaving the option of subsequent treatment open, such as cartilage transplantation procedures [8].

Zellner et al. [36] investigated the clinical and radiological regeneration of large and deep osteochondral defects of the knee. This was accomplished through the utilization of bone augmentation in conjunction with matrix‐guided autologous chondrocyte transplantation. The study demonstrated a comparable IKDC score of 80.3 at 3 years postsurgery and a superior MOCART score of 82.6 at 1‐year postsurgery. In comparison to our findings, Zellner et al. reported a notably higher MOCART score at 1‐year postsurgery. This discrepancy may be attributed to differences in surgical techniques, as their study utilized bone augmentation in combination with matrix‐guided autologous chondrocyte transplantation, which may have enhanced defect healing and cartilage regeneration. In contrast, our approach focused on fragment refixation with additional augmentation in only few cases. These differences highlight the potential influence of surgical techniques on radiological outcomes and suggest that further research is needed to directly compare these methods and evaluate their long‐term effectiveness.

It has been demonstrated that the refixation of unstable OD fragments using small‐fragment screws results in favourable clinical and radiological outcomes. The maintenance of the original hyaline cartilage confers a notable advantage over cartilage transplantation procedures [31], thereby establishing refixation as the preferred initial surgical approach. This is particularly relevant since, in the event of therapeutic failure, alternative therapeutic approaches, such as ACI, remain viable options [8]. The use of small metallic fragment screws for refixation ensures immediate and stable fixation, offering superior mechanical strength and stability compared to bioresorbable materials. This advantage is of particular significance in the context of larger or highly unstable fragments, where robust fixation is essential to prevent displacement and facilitate successful reintegration. Nevertheless, the necessity for a second surgical intervention to remove the screws represents a potential disadvantage that must be considered during the planning of treatment [20].

## LIMITATIONS

The present study is limited by its relatively short follow‐up period, which may not capture long‐term outcomes, as well by its retrospective study design. Additionally, the study's sample size is relatively modest, which may restrict the applicability of the findings. As well, this study compares different age range of patients, as well as a different time point of follow‐up examination. For future studies it would be of interest to include a comparison to other fixation methods to evaluate the difference in cartilage integration, as well as clinical outcome parameters.

## CONCLUSION

The therapeutic intervention for managing larger lesions of OD within the knee yielded positive clinical and MR morphologic outcomes within our patient cohort. The refixation of OD using metal screws can be considered a safe procedure with low complication rate and a high RTS rate.

## AUTHOR CONTRIBUTIONS

All authors contributed to the study conception and design. Data collection and analysis were performed by Franziska L. Breulmann and Patrice Dominitz. Data analysis was additionally performed by Julian Mehl. The first draft of the manuscript was written by Franziska L. Breulmann and Patrice Dominitz and all authors commented on previous versions of the manuscript. All authors read and approved the final manuscript.

## CONFLICT OF INTEREST STATEMENT

Sebastian Siebenlist has received consultant fee payments from Arthrex GmbH, KLS Martin Group and medi GmbH & Co.KG unrelated to this study. Julian Mehl is consultant for Arthrex GmbH and Ormed GmbH. The remaining authors declare no conflict of interest.

## ETHICS STATEMENT

This monocentric retrospective study was approved by the Ethical Committee of the Technical University of Munich (2022‐678‐S‐SR). The participants of the study all declared an informed written consent.

## Data Availability

The data that support the findings of this study are available from the corresponding author upon reasonable request.
